# Supplementary material for: Engineering a local acid-like environment in alkaline medium for efficient hydrogen evolution reaction
Source: Nat Commun. 2022 Apr 19;13:2024. doi: 10.1038/s41467-022-29710-w (PMC9019087; doi:10.1038/s41467-022-29710-w)
Supplement: Supplementary file 1 — Supplementary Information [file 41467_2022_29710_MOESM1_ESM.pdf]

Supplementary Information for

Engineering a local acid-like environment in alkaline medium for efficient hydrogen evolution reaction

Hao Tan[1,§], Bing Tang[1,§], Ying Lu[1,§], Qianqian Ji[1], Liyang Lv[1], Hengli Duan[1], Na Li[1], Yao Wang[1], Sihua Feng[1], Zhi Li[1], Chao Wang[1,*], Fengchun Hu[1], Zhihu Sun[1,*] and Wensheng Yan[1,*]

[1]National Synchrotron Radiation Laboratory, University of Science and Technology of China, Hefei 230029, P. R. China

[§]These authors contributed equally: Hao Tan, Bing Tang, Ying Lu.

[*]email: chaowng@ustc.edu.cn, zhsun@ustc.edu.cn, ywsh2000@ustc.edu.cn

Figures

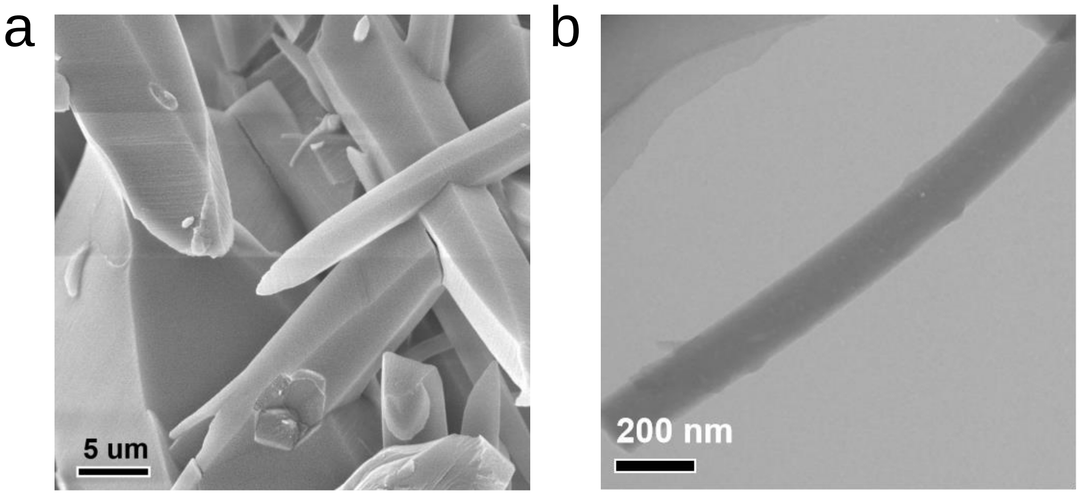

Supplementary Fig. 1. Morphology characterization. (a) SEM, (b) TEM images of MgMOF.

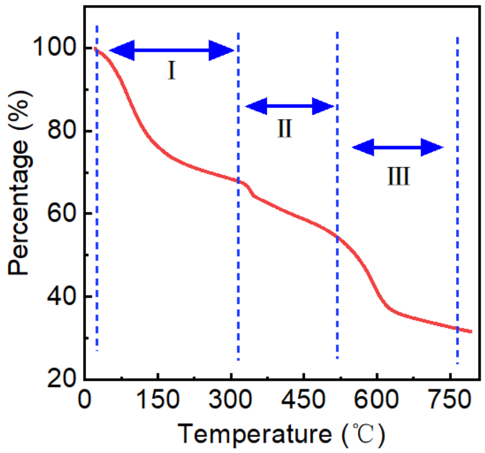

Supplementary Fig. 2. TGA analysis. TGA curve for the Pt/MOF.

The thermogravimetric analysis (TGA) curve in a mixture of nitrogen and oxygen revealed three weight loss steps. The first step of weight loss (25-310°C) could be attributed to the escape of water and solvent. the second weight loss (310°C-520°C)was caused by the decomposition of organic species leading to framework collapse. The third weight loss (520°C-800°C)was due to the formation of metal nanoparticles and inorganic oxides.

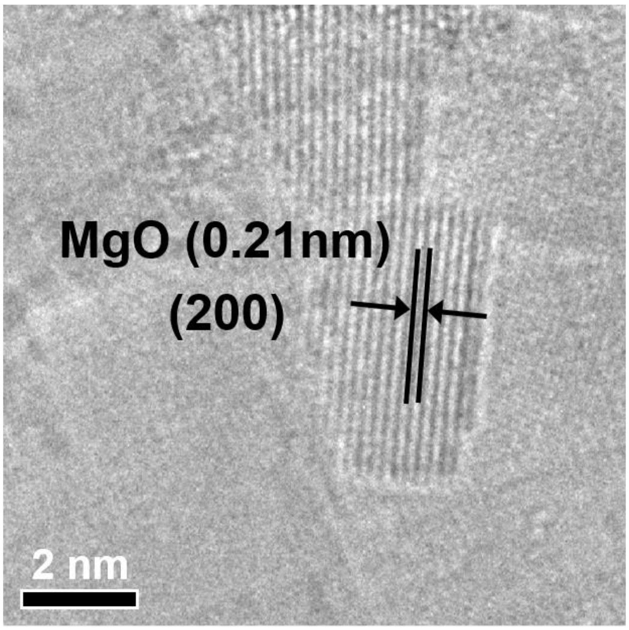

Supplementary Fig. 3. Crystal structure characterization. HRTEM image of MgO.

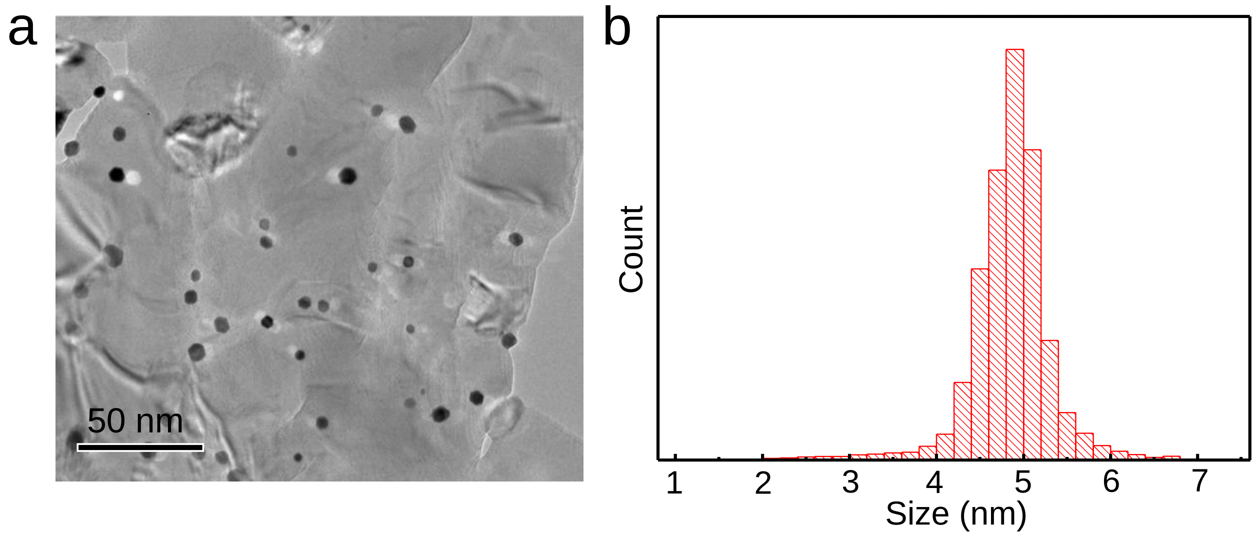

Supplementary Fig. 4. Catalyst size. TEM image (a) and corresponding particle Size Distribution Diagram (b) of Pt/MgO.

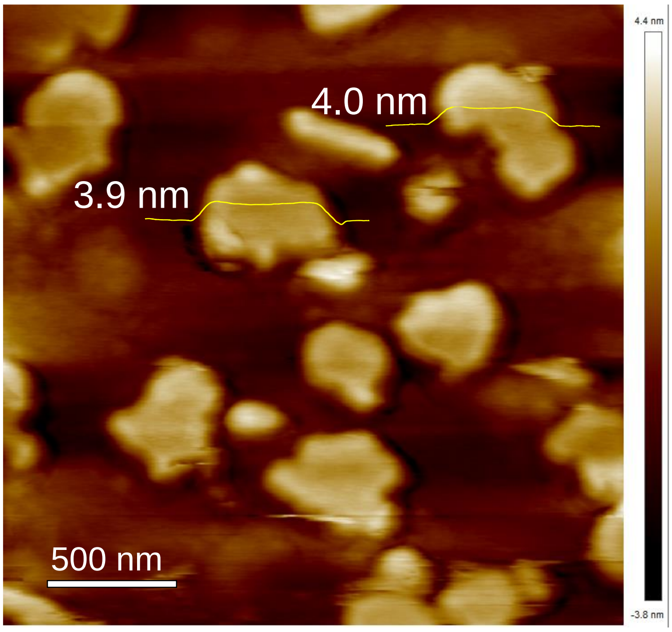

Supplementary Fig. 5. The thickness of Pt/MgO nanosheets. AFM image of Pt/MgO nanosheets.

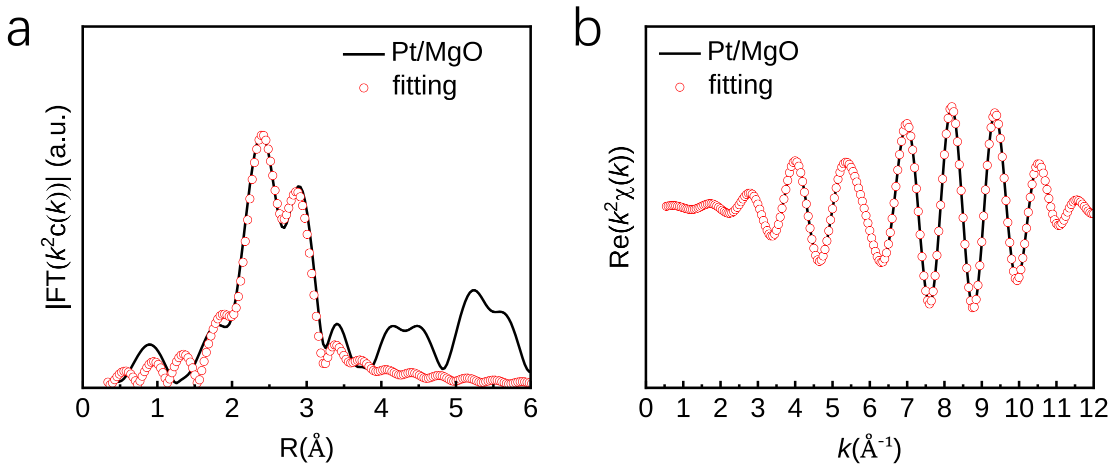

Supplementary Fig. 6. EXAFS analysis of Pt/MgO. k^2-weight FT-EXAFS fitting curves (a) and corresponding Re($k^2\chi(k)$) oscillations (b) of Pt/MgO at Pt K-edge.

**Sample**	**path**	**R / Å**	**N**	**σ^2 /(10^{-3} Å^2)**	**ΔE_0 / eV**
Pt/MgO	Pt-Pt	2.75±0.01	10.1±0.9	4.9±0.8	6.7±1.3

Supplementary Table 1. Pt/MgO EXAFS Curve Fitting Parameters.

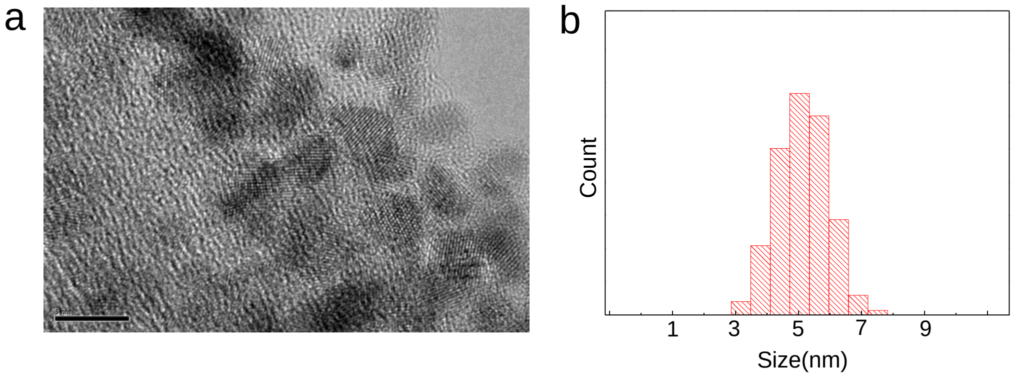

Supplementary Fig. 7. The size of Pt nanoparticles in Pt/C. TEM image (a) and corresponding particle Size Distribution Diagram (b) of Pt/C.

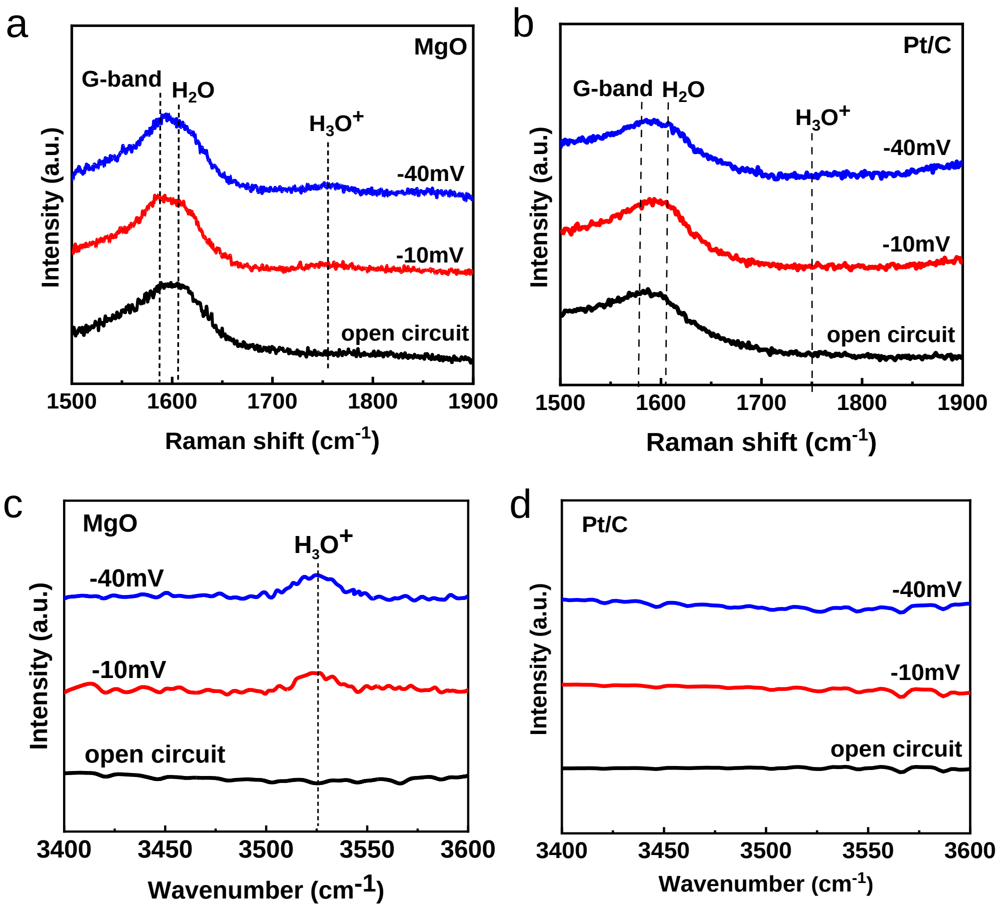

Supplementary Fig. 8. Operando spectroscopy characterization. The operando Raman spectra of MgO (a), and Pt/C (b), and the operando SR-FTIR of MgO (c) and Pt/C (d) in 1M KOH solution.

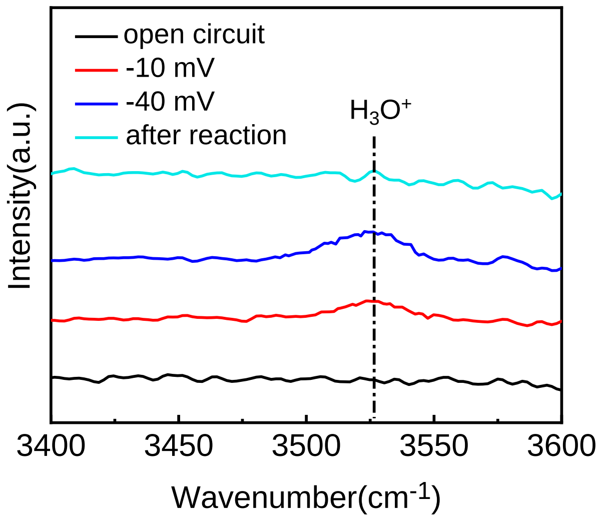

Supplementary Fig. 9. SR-FTIR spectra of Pt/MgO at various conditions. Operando infrared tests of Pt/MgO at different applied potential and after operando tests in 1M KOH solution.

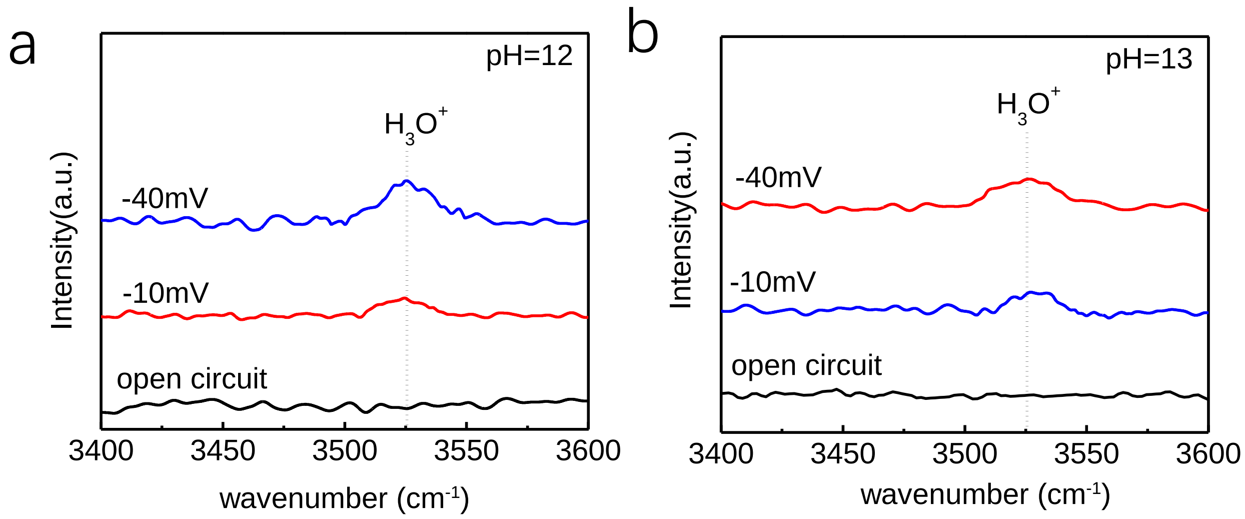

Supplementary Fig. 10. Operando SR-FTIR characterization at different alkaline environments. The operando SR-FTIR of Pt/MgO at pH=12 (a) and pH=13 (b), respectively.

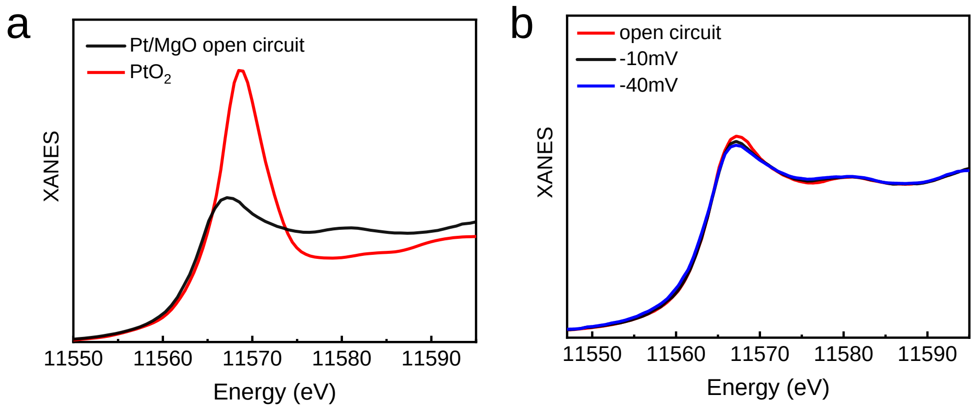

Supplementary Fig. 11. Ex-situ and in-situ XANES characterization. (a) Pt L_3-edge X-ray absorption near-edge structure (XANES) of Pt/MgO and PtO_2, (b) Operando Pt L_3-edge XANES spectra of Pt/MgO acquired in different conditions, and Pt/C was used for subtraction of difference XANES.

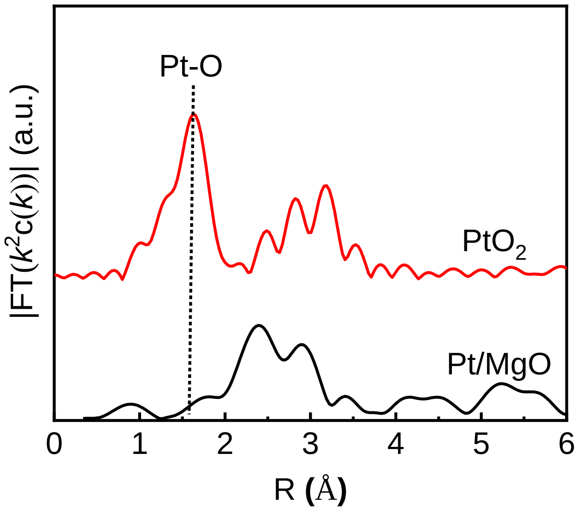

Supplementary Fig. 12. EXAFS analysis. The k^2-weighted Fourier transform (FT) spectra of Pt/MgO and PtO_2.

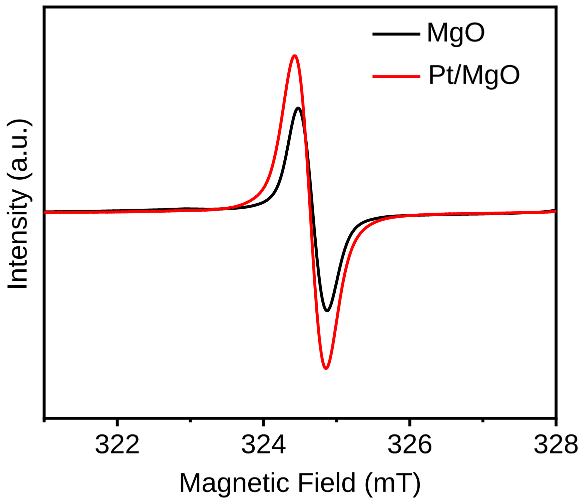

Supplementary Fig. 13. Oxygen vacancy characterization. The EPR spectra of MgO and Pt/MgO.

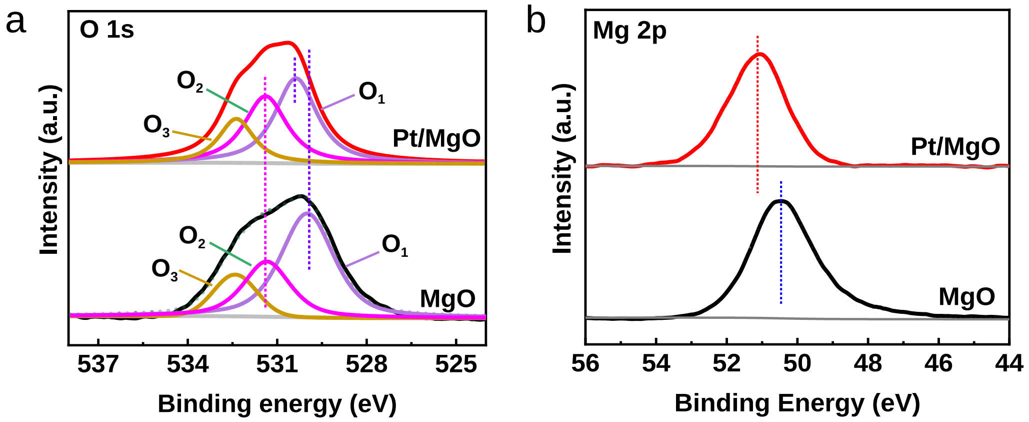

Supplementary Fig. 14. XPS analysis. The XPS spectras of O 1s (a) and Mg 2p (b) of MgO and Pt/MgO.

The O 1s spectra can be deconvoluted to three peaks at around 530.3 eV, 531.4 eV and 532.2 eV, originating from lattice oxygen, oxygen vacancies (O_V) and the surface oxidation, respectively.[1, 2] It can be noted that the position of lattice O shift to the higher binding energy side, and the Mg 2p spectra shows similar tendency, suggesting electrons transfer from MgO to the supported Pt nanoparticles.

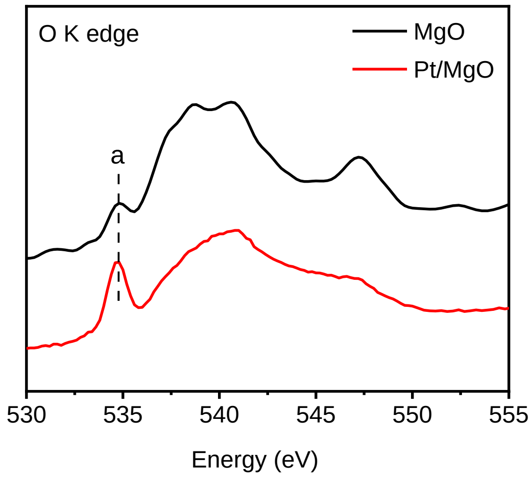

Supplementary Fig. 15. XANES analysis. O K-edge XANES spectras of MgO and Pt-MgO .

The peaks *a* correspond to the electron transition from oxygen p → Mg(1s)-O(2p) hybridized states.[3] Compared with MgO, the peak intensities of a increase after Pt embeded, this implies that the electrons in O 2p levels of Pt/MgO are decreased, which is consistent with the result of O 1s XPS.

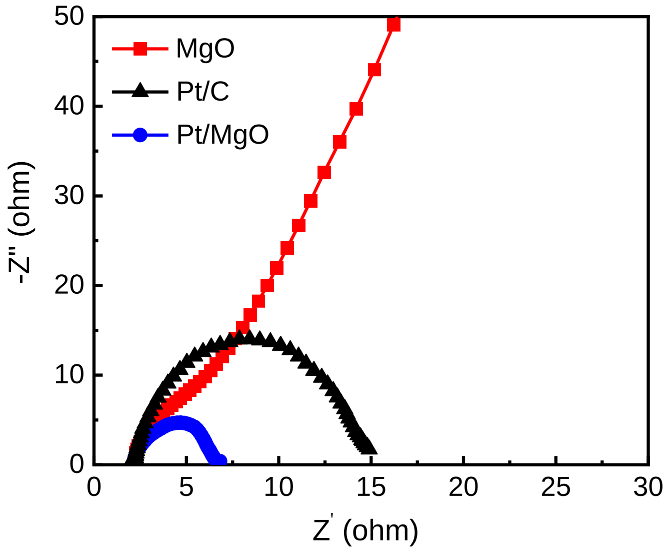

Supplementary Fig. 16. The Electrochemical Impedance Spectroscopy (EIS) analysis. The EIS curves of Pt/C, MgO and Pt-MgO.

The EIS curves of Pt/MgO shows an electrochemical resistance of 7 Ω, smaller than Pt/C (15 Ω), indicating the Pt/MgO can substantially facilitate the interfacial electron-transfer kinetics.

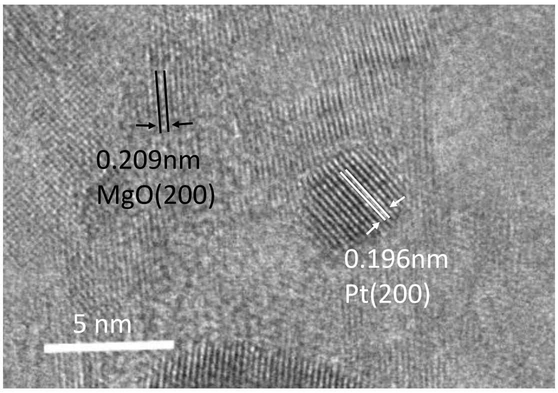

Supplementary Fig. 17. The atomic structure of Pt/MgO after reaction. The HRTEM image of Pt/MgO after the long-term test.

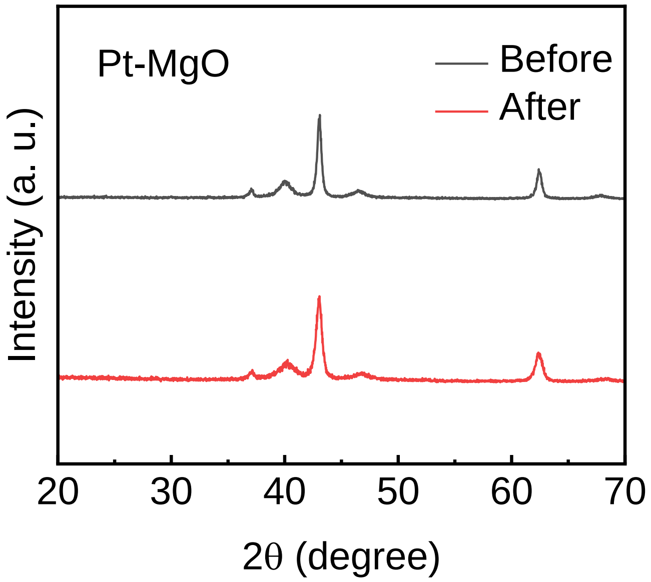

Supplementary Fig. 18. The atomic structure of Pt/MgO after reaction. The XRD spectra of Pt/MgO before and after the HER reaction.

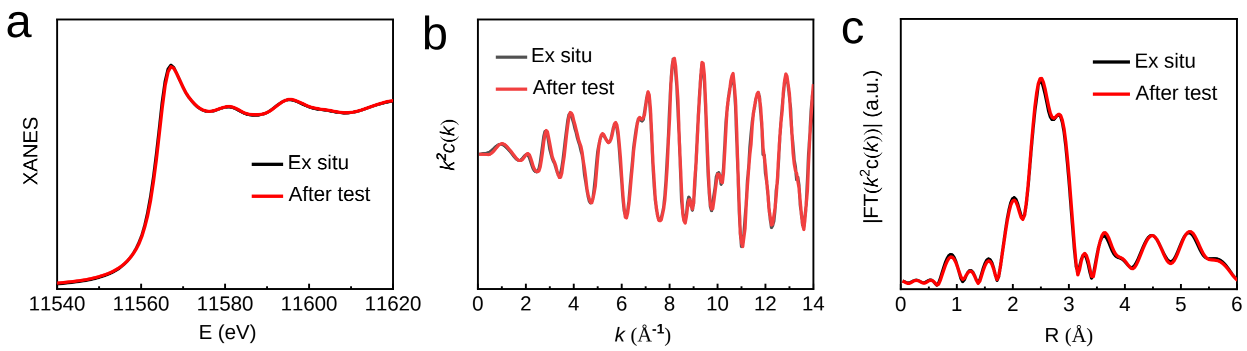

Supplementary Fig. 19. The fitting results of XAS spectra. (a) Pt L_3-edge XANES spectra, (b) corresponding $k^2\chi(k)$ oscillations, (c) Fourier transforms (FTs) of the Pt L_3-edge EXAFS oscillations of Pt/MgO before and after 20h chronopotentiometric tests at a current density of 10 mA cm^{-2}.

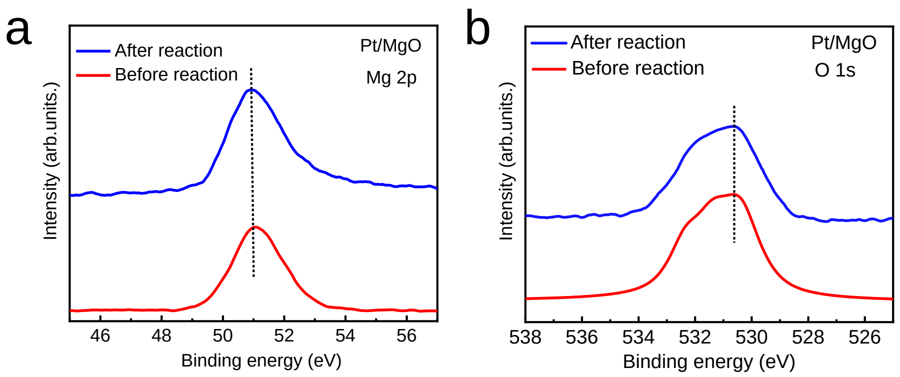

Supplementary Fig. 20. XPS analysis of Pt/MgO. The Mg 2*p* (a) and O 1*s* (b) XPS spectra of Pt/MgO before and after the long-term test.

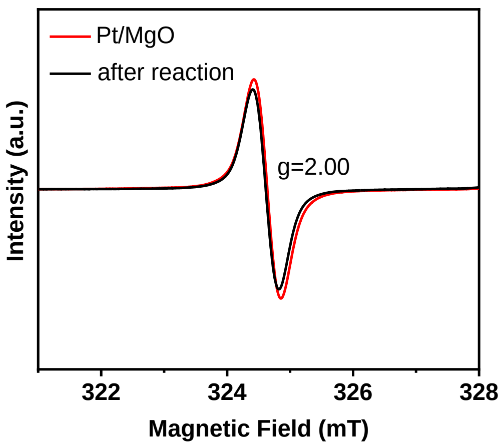

Supplementary Fig. 21. Oxygen vacancy characterization. The EPR spectra of Pt/MgO before and after the HER reaction.

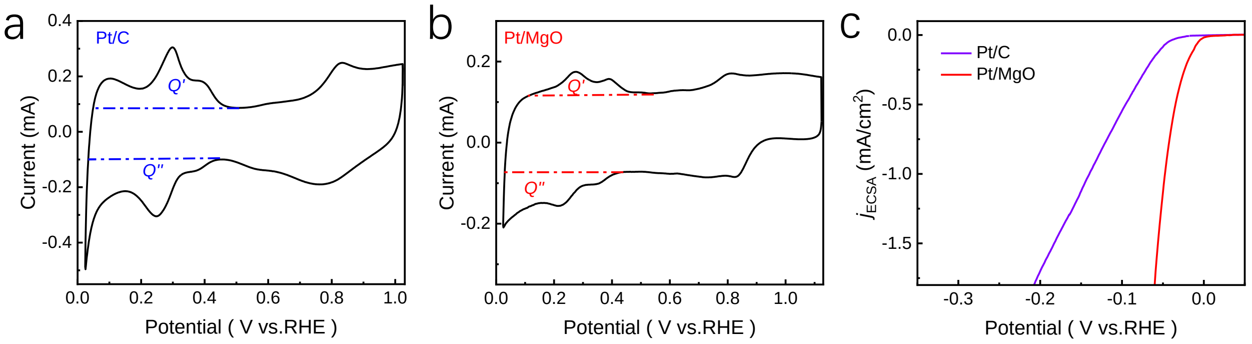

Supplementary Fig. 22. The cyclic voltammograms (CVs) used to determine the electrochemical surface area (ECSA). ECSA calculation of (a) Pt/C, (b) Pt/MgO in 1.0 M KOH. (c) ECSA-normalized HER polarization curves.

**Catalyst**	***Q'* *(mC)***	***Q"* *(mC)***	**Q_H (mC)**	**ECSA (cm^2)**
Pt/C	0.84	0.96	0.9	4.20
Pt/MgO	0.2	0.5	0.35	1.67

Supplementary Table 2. Q_H, *Q'*, *Q"* and ECSA of Pt/C and Pt/MgO.

ECSA of the Pt/C and Pt/MgO were determined from its CV curve in 1 M KOH electrolyte at the scan rate of 50 mV s^{-1}. As shown in Supplementary Fig. 22, the amounts of charge exchanged during the electro-adsorption (Q') and desorption (Q'') of H_2 on Pt can be calculated using following equation:

$$Q=\frac{1}{v}\int_{E1}^{E2} IdE$$

where *v* is the scan rate. The region contributed by the capacitive current from the double layer capacitance is deducted from the total charge. The coulombic charge of H_2 desorption (Q_H) on Pt catalysts can be calculated from the equation:

$$Q_H=\frac{1}{2}(Q' + Q'')$$

ECSA of Pt/C and Pt/MgO were calculated by the equation:

$$\text{ECSA}=\frac{Q_H}{0.21}$$

where the constant (0.21 mC·cm^{-2}) represents the charge required to oxidize a monolayer of H_2 on Pt. The charges Q_H, Q', Q'' and ECSA of Pt/C and Pt/MgO were summarized in Supplementary Table 2.

The ECSA-normalized HER polarization curves of the Pt/C and Pt/MgO are shown in Supplementary Fig. 22c. Evidently, Pt/MgO still has apparently larger current density than Pt/C, indicating the much higher intrinsic catalysis activity in Pt/MgO. Considering that the ECSA in Pt/MgO is lower than Pt/C, we conclude that the enhanced activity of HER in Pt/MgO is more possibly ascribed to the excellent intrinsic activity rather than to the increased ECSA.

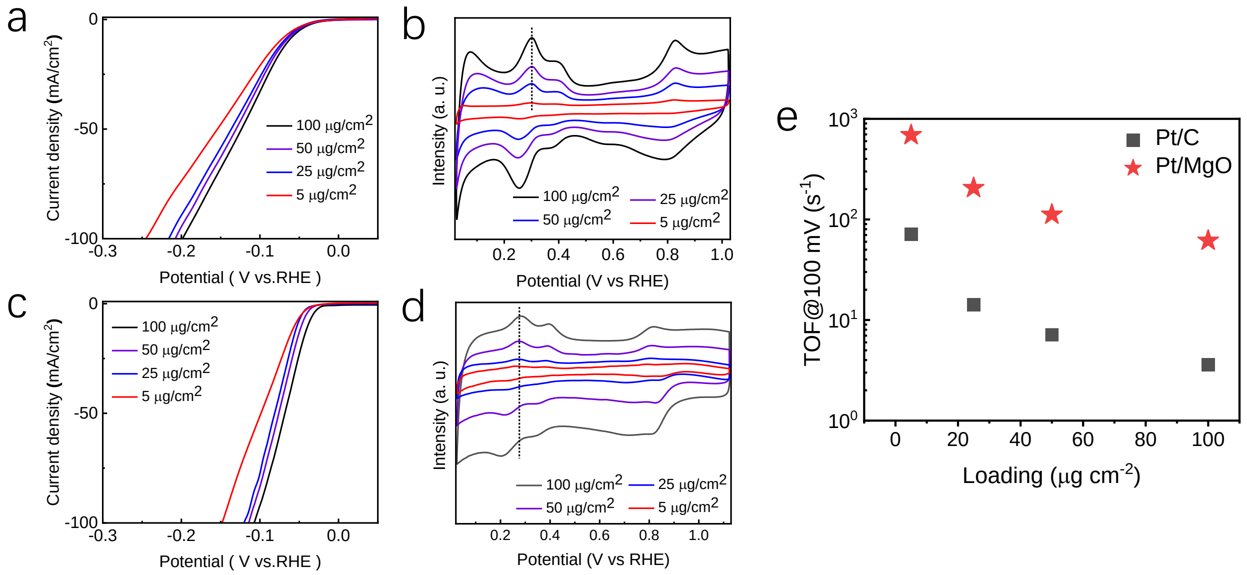

Supplementary Fig. 23. Intrinsic activity of the catalysts. Comparison of polarization curves (a) and cyclic voltammetries (b) for different Pt/C loading. Comparison of polarization curves (c) and cyclic voltammetries (d) for different Pt/MgO loading. (e) TOF at $\eta = -100$ mV vs Pt loading for all samples.

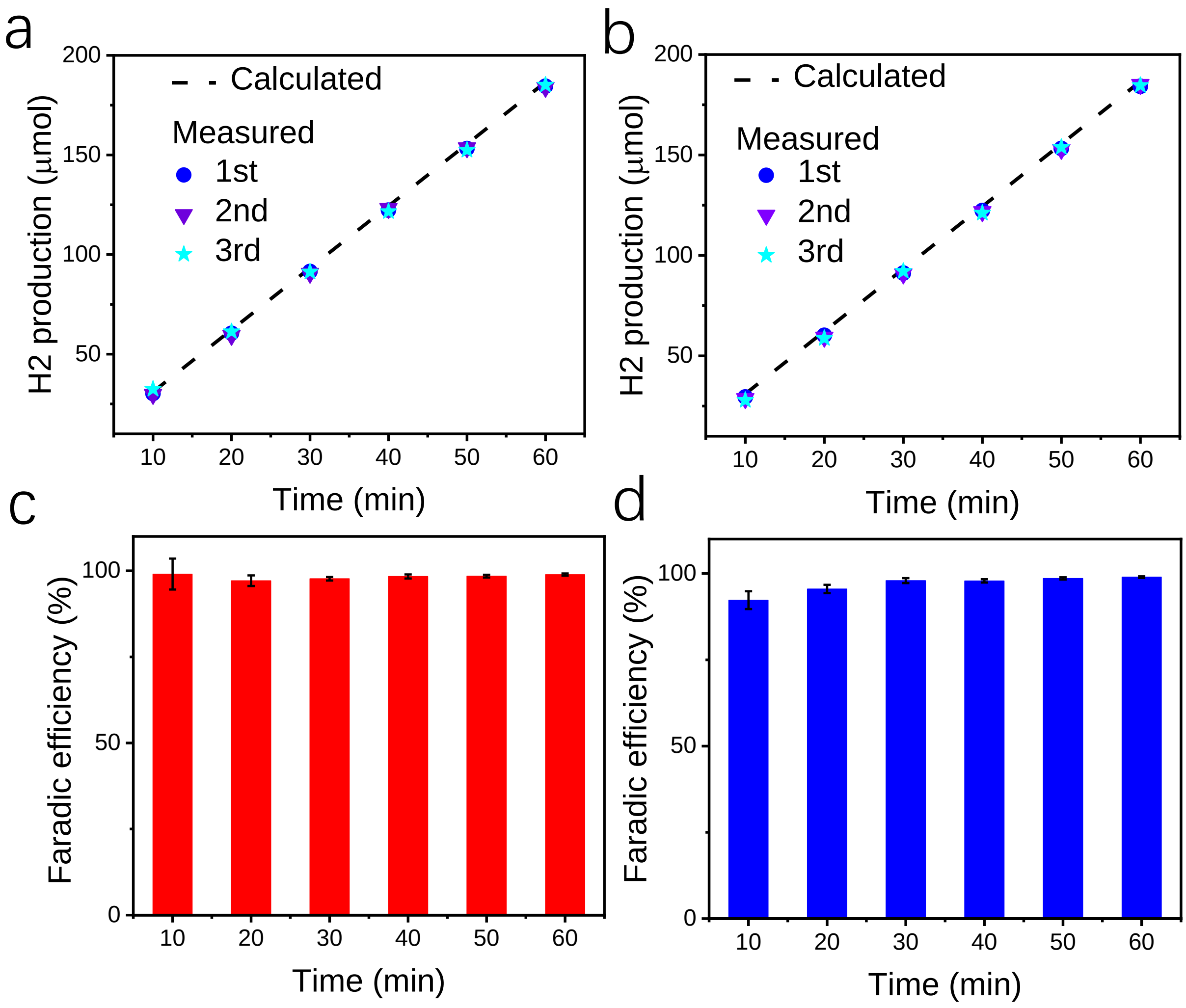

Supplementary Fig. 24. Faradic efficiency calculation. The amount of gas theoretically calculated and experimentally measured versus time over HER for Pt/MgO (a) and Pt/C (b), Faradic efficiency of the Pt/MgO (c) and Pt/C (d) for hydrogen. The error bars are defined by three independent measurements.

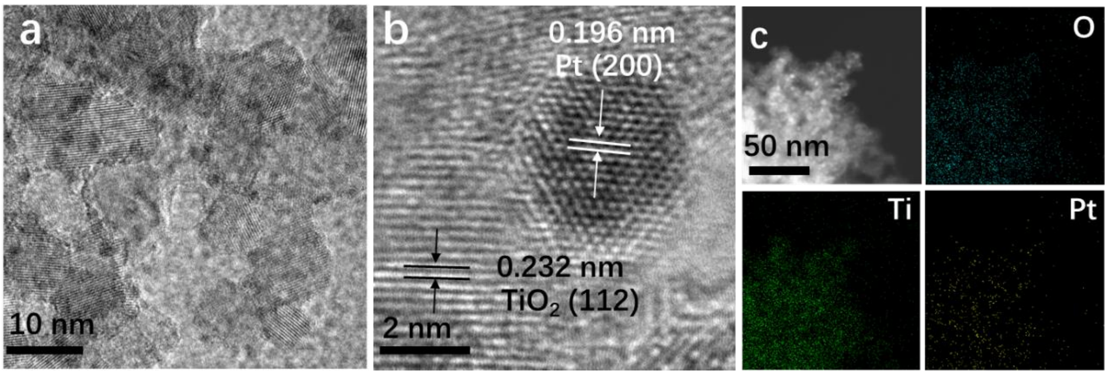

Supplementary Fig. 25. Structural and compositional characterizations**.** (a) TEM, (b) HRTEM, and (c) EDX mapping images of the Pt/TiO_2.

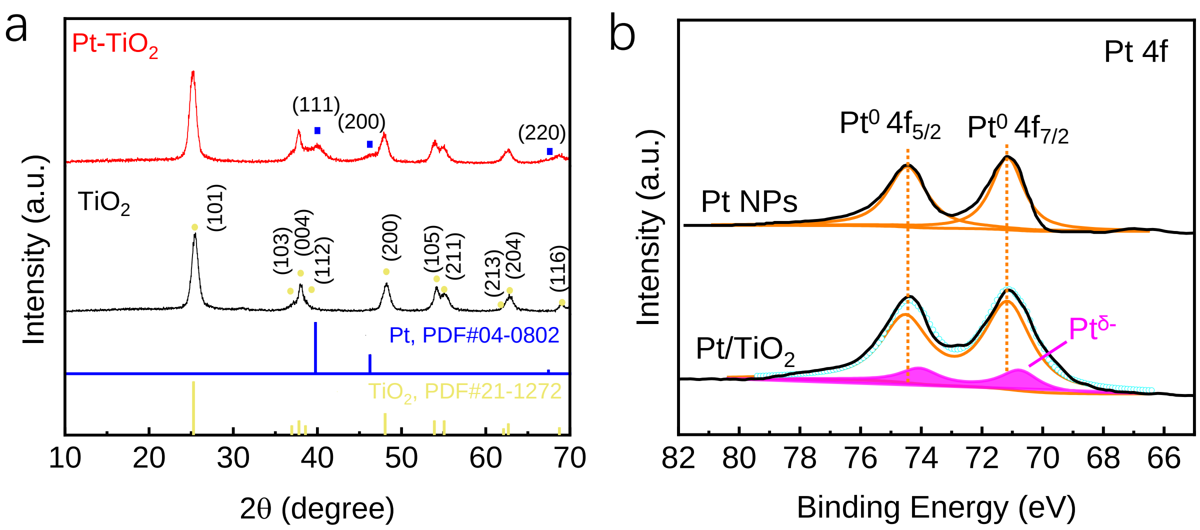

Supplementary Fig. 26. Atomic and electronic structure characterizations. (a) XRD pattern, and (b) XPS spectrum of the Pt/TiO_2.

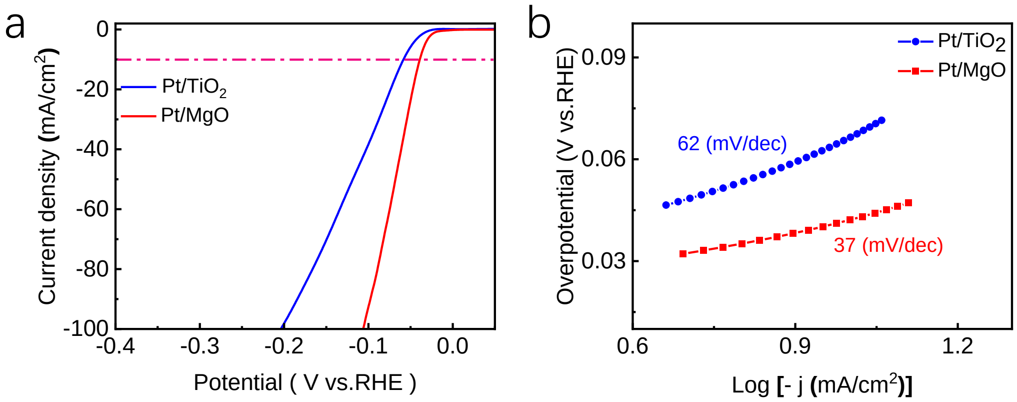

Supplementary Fig. 27. HER activity comparison. (a) Linear sweep voltammetry (LSV), (b) the corresponding Tafel plots calculated from the LSV curves of the Pt/TiO_2 and Pt/MgO.

To demonstrate that the negatively charged $Pt^{\delta-}$ alone is not enough for the improved HER catalysis, we firstly replace MgO by another substrate that can induce negative-charged $Pt^{\delta-}$ but has not strong H_2O

dissociation ability. Based on literature reports on the negative-charged $Au^{\delta-}$ and $Ni^{\delta-}$ on TiO_2, which also possesses the F centers, we used TiO_2 as the alternative support to induce the negatively-charged $Pt^{\delta-}$. As shown in Supplementary Fig. 25, we successfully obtained the Pt/TiO_2. Then we carried out the Pt 4f XPS measurements for the synthesized Pt/TiO_2. As expected, a peak at 70.6 eV is observed in the lower binding energy side than the Pt^0 state, suggesting the formation of negatively-charged $Pt^{\delta-}$ atoms. Using the in-situ Raman spectra, we demonstrate that the negatively-charged $Pt^{\delta-}$ could not create the acid-like environment. This implies that the water dissociation ability of negatively-charged $Pt^{\delta-}$ is not satisfactory, consistent with our calculation results. The LSV tests as shown in Supplementary Fig. 27 also indicate the relatively poor HER activity of $Pt^{\delta-}$/TiO_2, with an overpotential of 60 mV at the current density of 10 mA cm^{-2}. The significantly lower activity of $Pt^{\delta-}$/TiO_2 than $Pt^{\delta-}$/MgO leads us to conclude that the negatively-charged $Pt^{\delta-}$ species could not be responsible for all the improvement on HER performance.

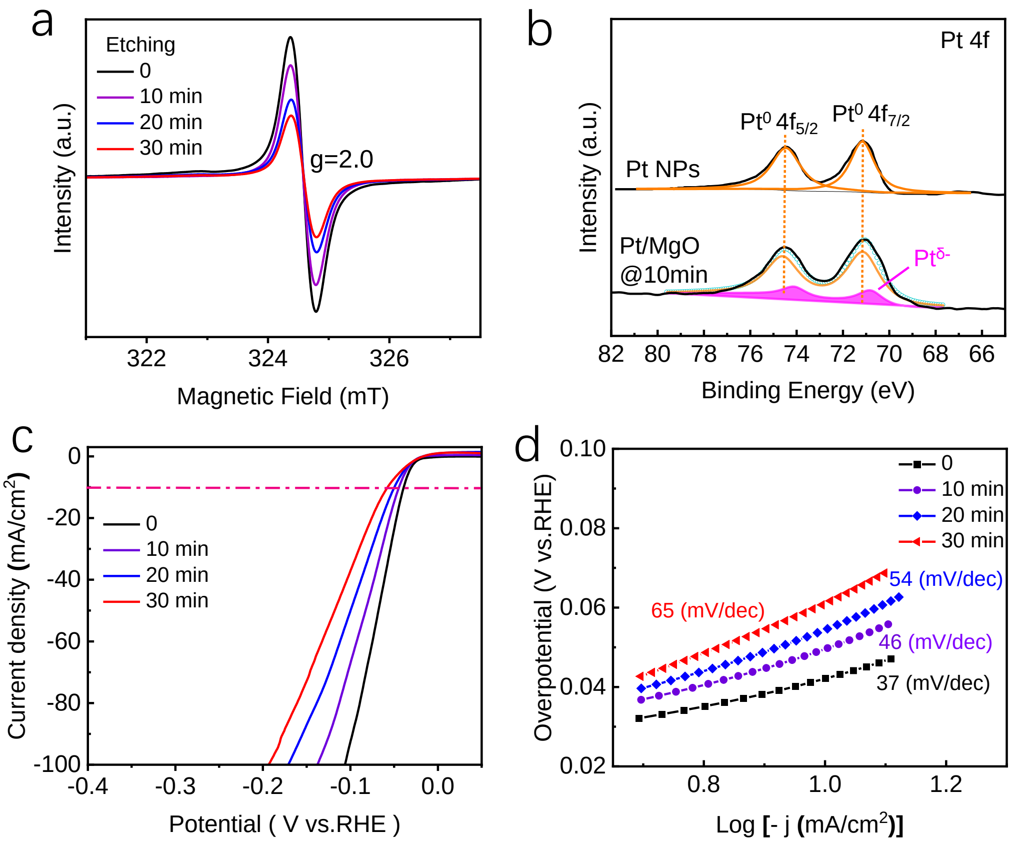

Supplementary Fig. 28. Atomic and electronic structure of Pt/MgO samples with different acid-etching time and their HER activity. (a) The EPR spectra, (b) the XPS spectra, (c) Linear sweep voltammetry (LSV) curves, and (d) the corresponding Tafel plots calculated from the LSV curves of the Pt/MgO samples with different with etching times.

we used the diluted acid (10μM H_2SO_4) etching strategy to gradually decrease the amount of oxygen vacancies in Pt/MgO. The HER property of Pt/MgO becomes worse with the reduced oxygen vacancies, indicating the important role of V_O-MgO in catalyzing HER. Moreover, after 30 min of acid-etching treatment, the Tafel slope increases from 37 to 65 mV dec^{-1}, indicating the changed rate-determining step of HER from H_2 desorption to the water dissociation. The Pt 4f XPS measurements for the Pt/MgO@10min of acid-etching show that the Pt still maintains its negatively charged state. These results further reinforce our claim that the better catalytic activity does not just come from the modification of Pt. The local acid-like environment created by the strong water dissociation ability of MgO and the proton aggregation ability of negative valence of Pt are the keys to the high HER activity.

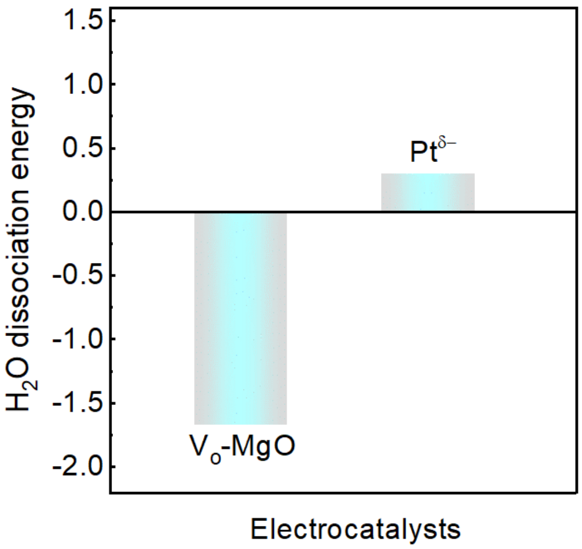

Supplementary Fig. 29. The water dissociation ability. The water dissociation energy of MgO and negatively charged Pt.

We also established the theoretical model for calculating the H_2O dissociation energy on the negatively charged Pt sites, and compare it with that of V_O-MgO, as shown in Supplementary Fig. 29. The energy barrier for the dissociation of H_2O into OH and H on negatively charged Pt site is 0.34 eV, significantly higher than V_O-MgO (-1.7 eV). Such a water dissociation ability of negatively charged Pt is similar to the Pt/C, however, the electrocatalytic HER performances of our V_O-MgO/Pt significantly superior to the Pt/C, due to the rate-determining step of Pt/C is the dissociation of H_2O. These results imply the modification of Pt cannot boosts such a high alkaline HER activity.

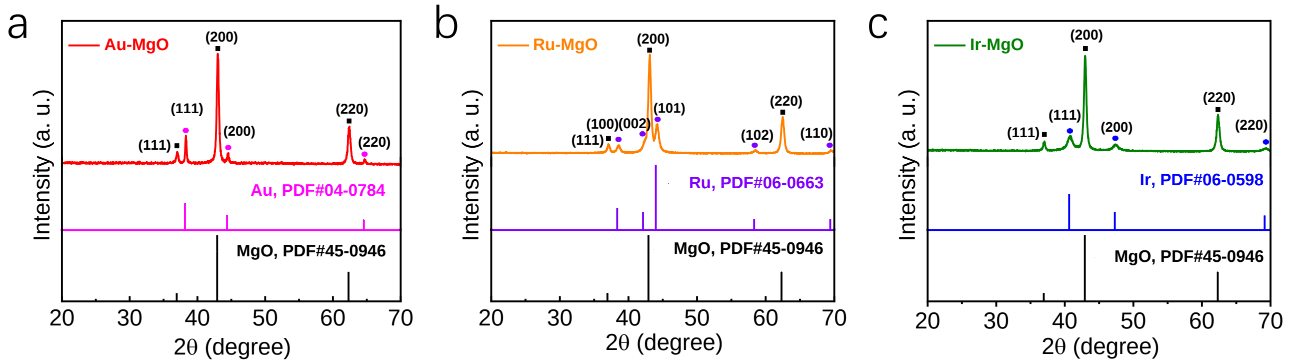

Supplementary Fig. 30. XRD patterns of different samples. XRD patterns of the (a)Au/MgO, (b) Ru/MgO and (c) Ir/MgO.

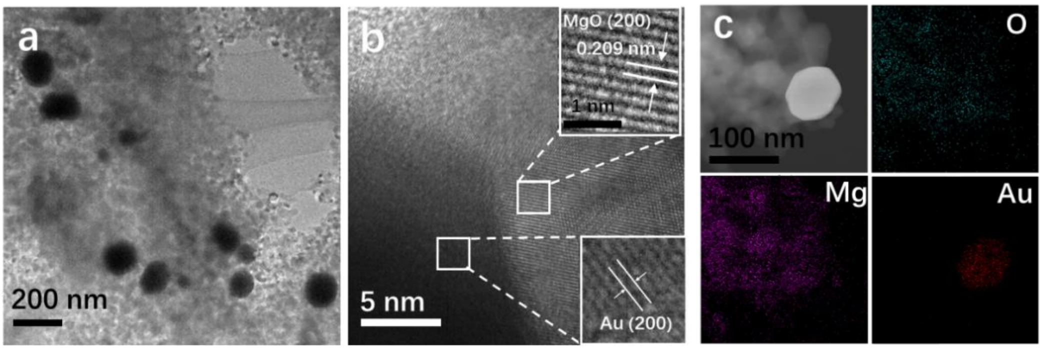

Supplementary Fig. 31. Structural and compositional characterizations. (a) TEM image, (b) HRTEM, and (c) EDX mapping images of the Au/MgO.

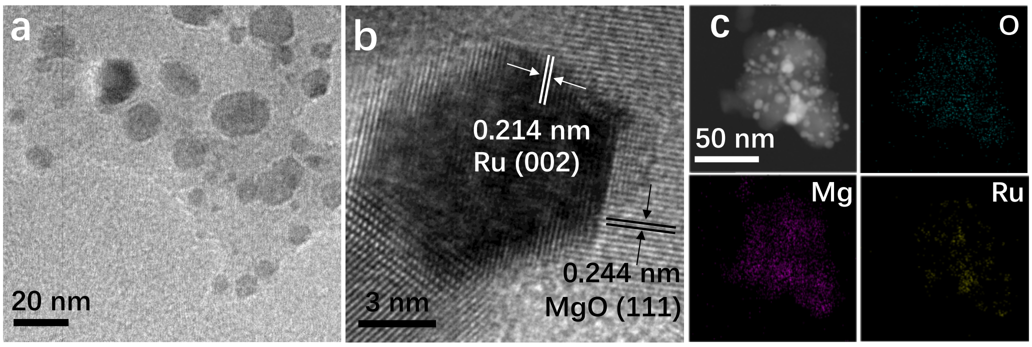

Supplementary Fig. 32. Structural and compositional characterizations. (a) TEM image, (b) HRTEM, and (c) EDX mapping images of the Ru/MgO.

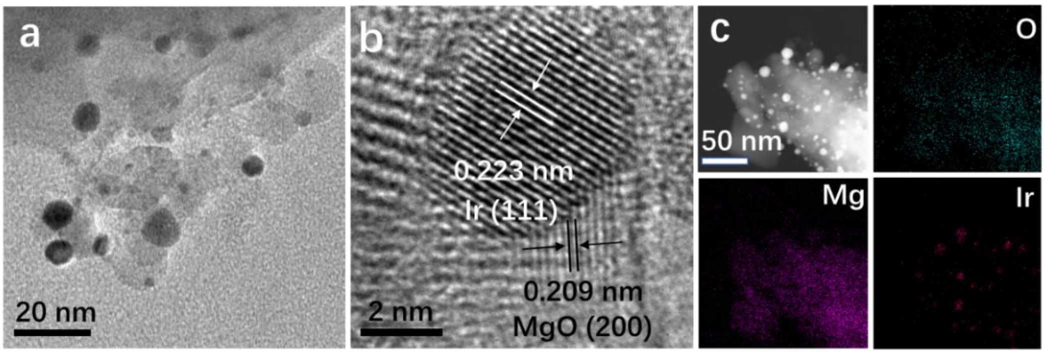

Supplementary Fig. 33. Structural and compositional characterizations. (a) TEM image, (b) HRTEM, and (c) EDX mapping images of the Ir/MgO.

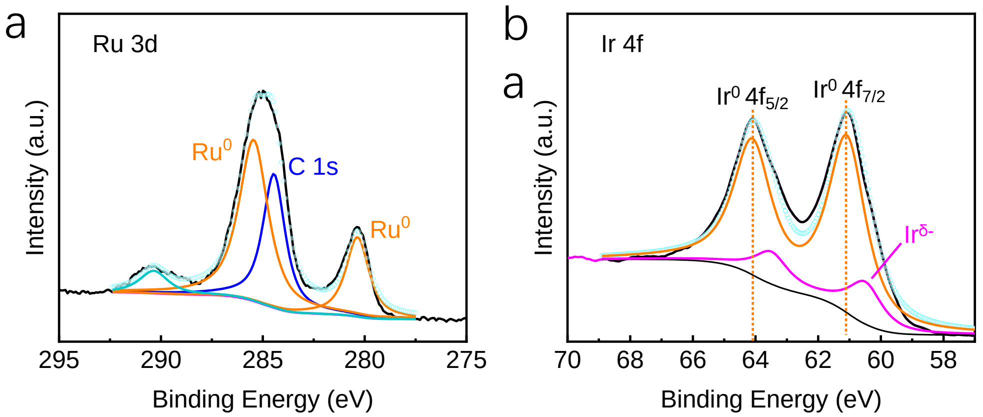

Supplementary Fig. 34. XPS analysis. XPS spectrums of the (a) Ru/MgO and (b)Ir/MgO.

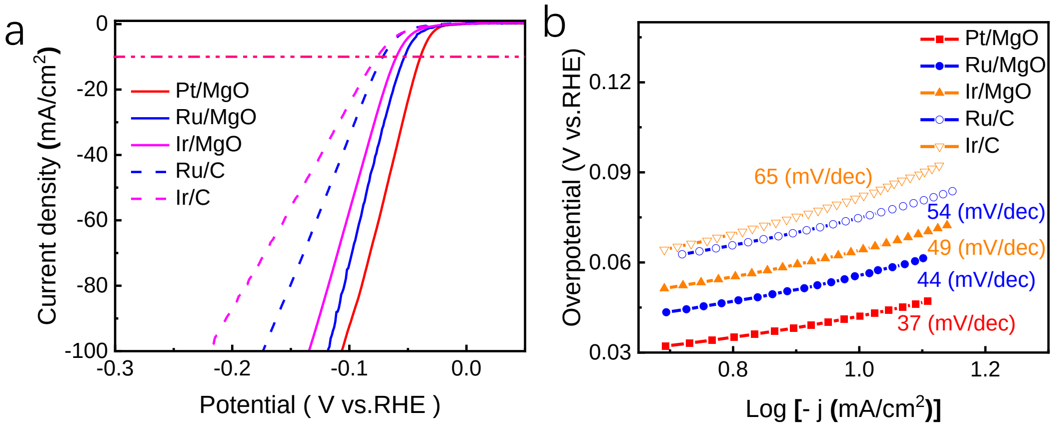

Supplementary Fig. 35. Comparison of electrochemical HER parameters. (a) Linear sweep voltammetry (LSV) curves, (b) the corresponding Tafel plots calculated from the LSV curves of the Pt/MgO, Ru/MgO, Ir/MgO, Pt/C, Ru/C and It/C.

Then, we tried to used other noble metal, Au and Ru, to replace the Pt. By using the same synthesis steps, the Au/MgO and Pt/MgO are obtained and their XRD patterns are shown in Supplementary Fig. 30. It's obvious that we successfully obtained the Au/MgO and Ru/MgO. Then we took their TEM images. Unfortunately, the size of the Au is too large (~100nm), it is not suitable for later research. In contrast, the size of the nanoparticle of Ru and Ir are ~20nm and ~10 nm, respectively (Supplementary Fig. 31-33). XPS spectrums showed that negative valence species were formed on the Ir surface but not on Ru (Supplementary Fig. 34). Then compared with Ru/C and Ir/C, the HER performance of the Ru/MgO and Ir/MgO is much better (Supplementary Fig. 35), indicating that an abundant of H^+ created by MgO could improve the alkaline HER for other noble metal HER catalyst. However, the HER performance is still not as good as Pt/MgO. This is due to that Pt have the most approachable zero hydrogen absorption energy. Besides, the negative-charged $Pt^{\delta-}$ is also beneficial for the H_3O^+ accumulation, which is evidenced by operando XANES.

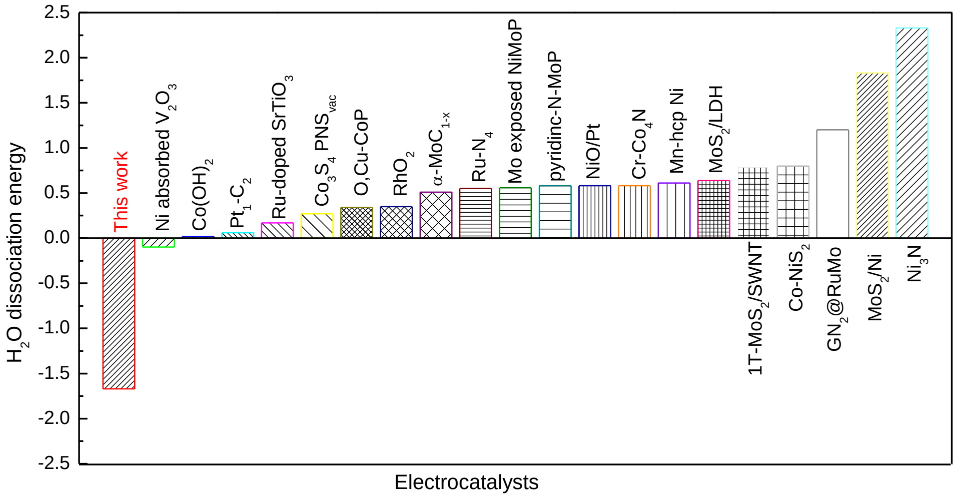

Supplementary Fig. 36. Comparison of water dissociation ablitity. Summary of the water dissociation energy of different samples.

Including the Ni-absorbed V_2O_3 (-0.099eV)[4], $Co(OH)_2$ (0.02eV)[5], Pt_1-C_2(0.06eV)[6], Ru-doped $SrTiO_3$(0.17)[7], Co_3S_4 PNS_{vac}(0.27 eV)[8], O, Cu–CoP(0.34eV)[9], RhO2(0.35eV)[10], α-MoC_{1-x}(0.51 eV)[11], Ru–N_4(0.550 eV)[12], Mo exposed NiMoP(0.56 eV)[13], pyridinic–N–MoP(0.58 eV)[14], NiO/Pt(0.58 eV)[15], Cr–Co_4N (0.582eV)[16], Mn-hcp Ni (0.61eV)[17], MoS_2/LDH(0.64 eV)[18], 1T-MoS_2/SWNT(0.79 eV)[19], Co-NiS_2 (0.80 eV)[20], GN_2@RuMo(1.2eV)[21], MoS_2/Ni$(OH)_2$ (1.83 eV)[22], Ni_3N(2.33 eV)[23].

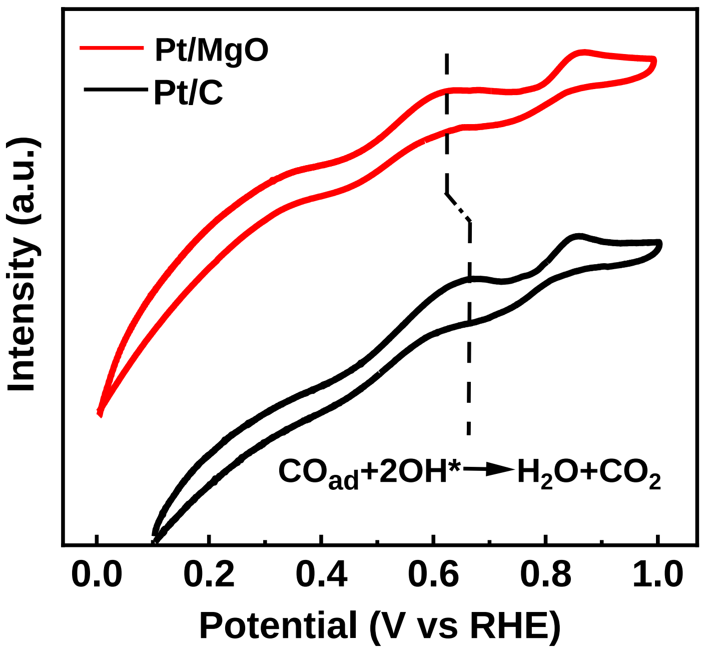

Supplementary Fig. 37. The bonding strength of OH_{ad}. CO stripping voltammetry curves for Pt/MgO, Pt/C in 1M KOH.

CO stripping measurements were also carried out to monitor the bonding strength of OH_{ad} at the called "butterfly region" region, which is generally assumed to represent the formation of hydroxyl species (OH_{ad}).[24] As shown in Supplementary Fig. 37, the stripping peak for CO_{ad} oxidation of Pt/MgO is shifted toward relatively low potential indicates stronger interaction between OH_{ad} and catalyst, indicating better H_2O dissociation proceeds. As a result, the Pt/MgO catalyst performs an optimal H*/OH* adsorption. For Pt/C catalyst, the water dissociation as a Volmer step requires high activation energy to drive the reaction, which leads to the sluggish production of H_{ad} and slows down the reactivity. In short, the Pt/MgO catalyst creates a local acid-like environment in alkaline solution, which provides Pt with a favorable reaction environment for HER in alkaline condition.

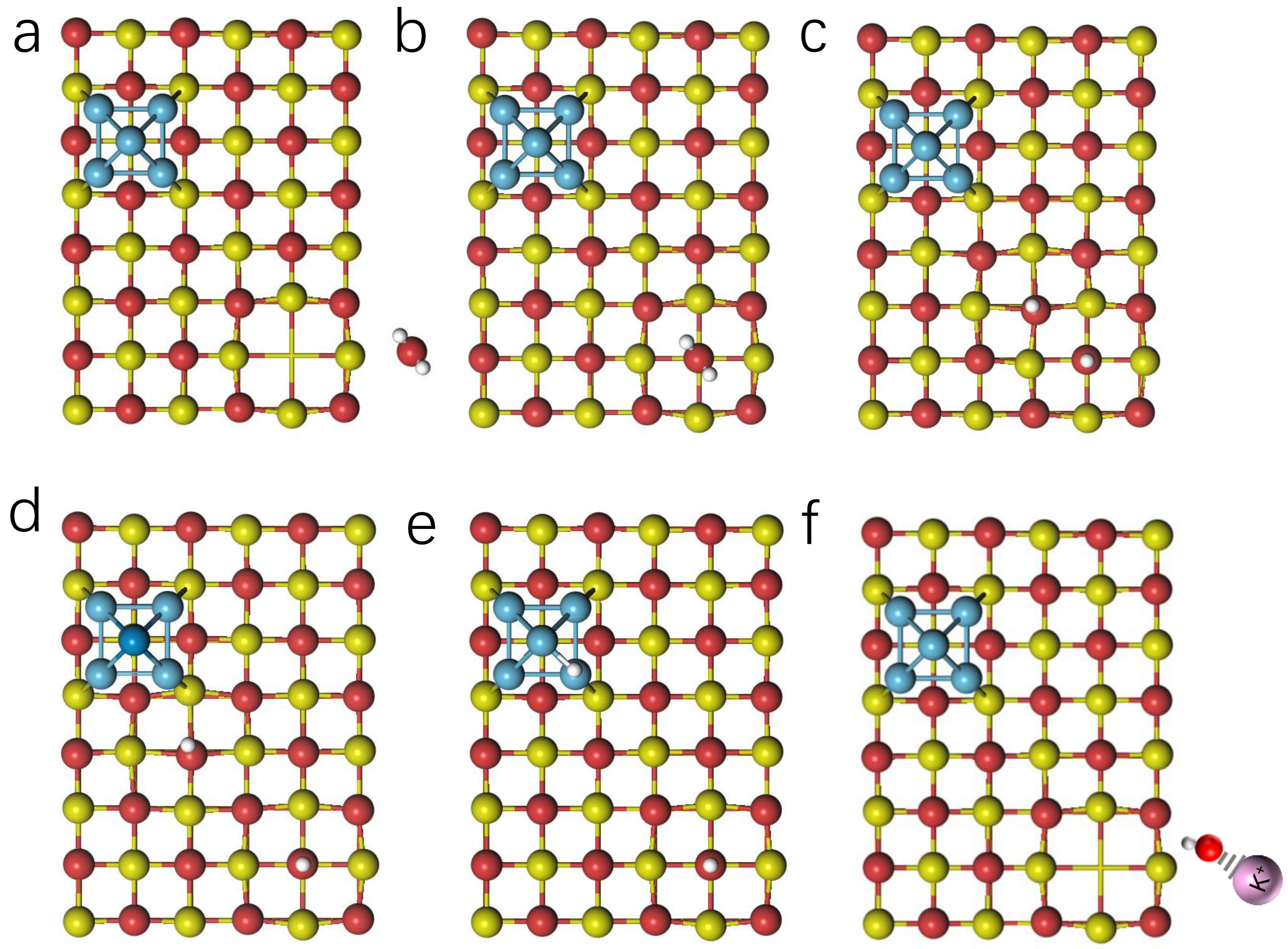

Supplementary Fig. 38. DFT calculation models. Computational models of the (a) Pt/MgO nanosheets, (b) H_2O absorption, (c)-(e) H_3O^+ migration. (f) OH^- desorption with K^+ assistant, and the calculated desorption energy is -4.242 eV.

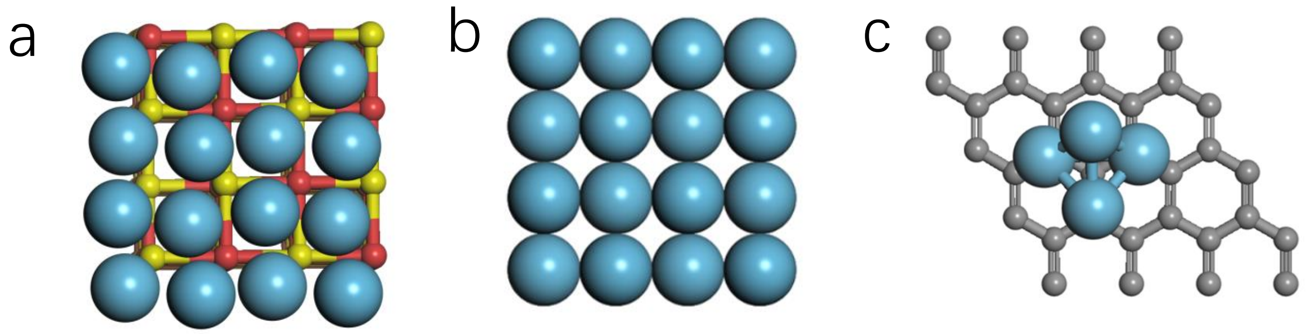

Supplementary Fig. 39. DFT calculation models. Atomic structures of $Pt^{\delta-}$ (a), Pt^{0} (b) and $Pt^{\delta+}$ (c) model catalysts.

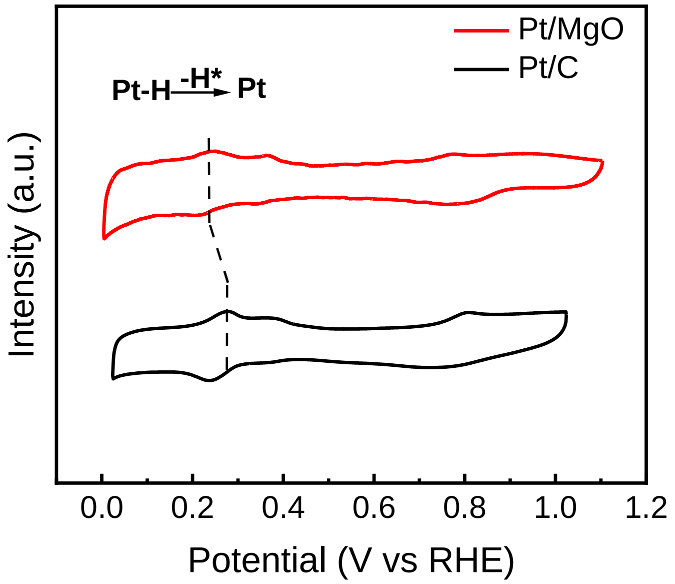

Supplementary Fig. 40. The adsorption energy of intermediates. Cyclic voltammetry (CV) curves of Pt/MgO, Pt/C in 1M KOH.

Since the adsorption energy of intermediates during HER process under alkaline environment dominates the reaction activity, it is of great significance to explore the interaction between OH*, H* and electrocatalyst.[25, 26] thus we examined the cyclic voltammograms (CVs) to study the interaction between H* and the catalytic surface. For Pt/MgO, it was observed that the under-potential deposition H (H_{upd}) peak shifted to a lower potential in comparison to Pt/C. The relatively negative potential manifests the weakened H adsorption, which is conductive to the fast evolution of H_2 contributing to the enhanced alkaline HER performance in 1M KOH.

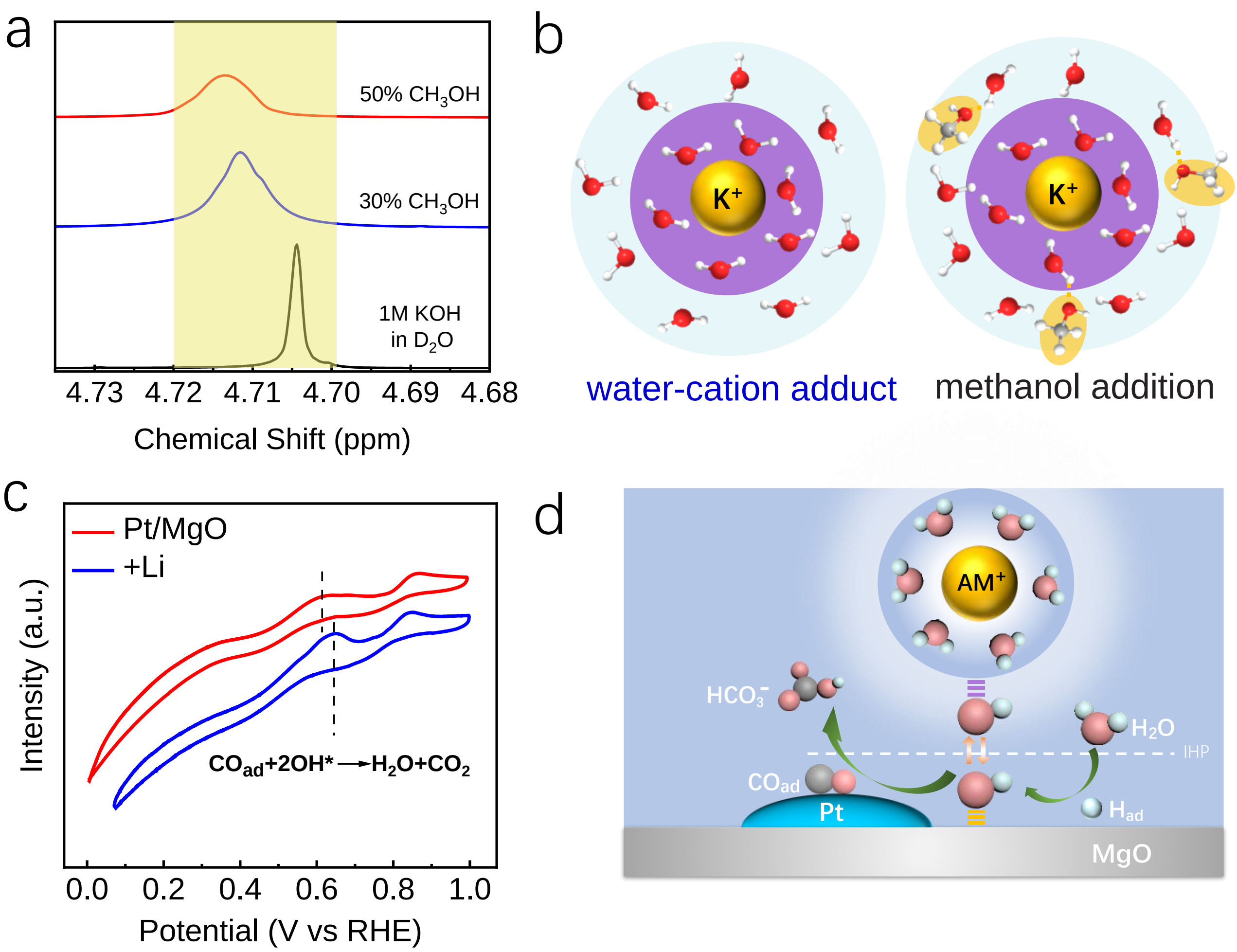

Supplementary Fig. 41. Non-covalent interactions and schematic representation of the double-layer structure. (a) ^{2}H NMR spectra, (b) Schematic of changes in the K^+-water adduct, together with methanol addition. (c) CO stripping (inset) of Pt in 1 M KOH and plus $LiClO_4$. (d) Schematic illustration of HER and CO oxidation mechanism.

Supplementary References

1. Xu M*, et al.* TiO_{2-x}–Modified Ni Nanocatalyst with Tunable Metal–Support Interaction for Water–Gas Shift Reaction. *ACS Catal.* **7**, 7600-7609 (2017).

2. Kim JH, Jang YJ, Kim JH, Jang JW, Choi SH, Lee JS. Defective $ZnFe_2O_4$ nanorods with oxygen vacancy for photoelectrochemical water splitting. *Nanoscale* **7**, 19144-19151 (2015).

3. Luches P*, et al.* X-ray absorption study at the Mg and O K edges of ultrathin MgO epilayers on Ag (001). *Physical Review B* **69**, 045412 (2004).

4. Yang CF*, et al.* Ni-Activated Transition Metal Carbides for Efficient Hydrogen Evolution in Acidic and Alkaline Solutions. *Adv. Energy Mater.* **10**, 10 (2020).

5. Luo YT*, et al.* Two-Dimensional MoS_2 Confined $Co(OH)_2$ Electrocatalysts for Hydrogen Evolution in Alkaline Electrolytes. *ACS Nano* **12**, 4565-4573 (2018).

6. Fang S*, et al.* Uncovering near-free platinum single-atom dynamics during electrochemical hydrogen evolution reaction. *Nat. Commun.* **11**, 1029 (2020).

7. Dai J*, et al.* Single-phase perovskite oxide with super-exchange induced atomic-scale synergistic active centers enables ultrafast hydrogen evolution. *Nat. Commun.* **11**, 5657 (2020).

8. Zhang C, Shi YM, Yu YF, Du YH, Zhang B. Engineering Sulfur Defects, Atomic Thickness, and Porous Structures into Cobalt Sulfide Nanosheets for Efficient Electrocatalytic Alkaline Hydrogen Evolution. *ACS Catal.* **8**, 8077-8083 (2018).

9. Xu K*, et al.* Yin-Yang Harmony: Metal and Nonmetal Dual-Doping Boosts Electrocatalytic Activity for Alkaline Hydrogen Evolution. *ACS Energy Letters* **3**, 2750-2756 (2018).

10. Li Z*, et al.* Stable Rhodium (IV) Oxide for Alkaline Hydrogen Evolution Reaction. *Adv. Mater.* **32**, 7 (2020).

11. Baek D*, et al.* Ordered Mesoporous Metastable alpha-MoC_{1-x} with Enhanced Water Dissociation Capability for Boosting Alkaline Hydrogen Evolution Activity. *Adv. Funct. Mater.* **29**, 8 (2019).

12. Hu C*, et al.* Partial-Single-Atom, Partial-Nanoparticle Composites Enhance Water Dissociation for Hydrogen Evolution. *Adv. Sci.* 2001881 (2020).

13. Yu L*, et al.* Ternary $Ni_{2(1-x)}Mo_{2x}P$ nanowire arrays toward efficient and stable hydrogen evolution electrocatalysis under large-current-density. *Nano Energy* **53**, 492-500 (2018).

14. Zhao D*, et al.* Synergistically Interactive Pyridinic-N-MoP Sites: Identified Active Centers for Enhanced Hydrogen Evolution in Alkaline Solution. *Angew. Chem. Int. Edit.* **59**, 8982-8990 (2020).

15. Chen ZJ*, et al.* Highly Dispersed Platinum on Honeycomb-like NiO@Ni Film as a Synergistic Electrocatalyst for the Hydrogen Evolution Reaction. *ACS Catal.* **8**, 8866-8872 (2018).

16. Yao N*, et al.* Synergistically Tuning Water and Hydrogen Binding Abilities Over Co4N by Cr Doping for Exceptional Alkaline Hydrogen Evolution Electrocatalysis. *Adv. Energy Mater.* **9**, 8 (2019).

17. Shao Q*, et al.* Stabilizing and Activating Metastable Nickel Nanocrystals for Highly Efficient

Hydrogen Evolution Electrocatalysis. *ACS Nano* **12**, 11625-11631 (2018).

18. Xiong P*, et al.* Interface Modulation of Two-Dimensional Superlattices for Efficient Overall Water Splitting. *Nano letters* **19**, 4518-4526 (2019).

19. Cao DF*, et al.* Engineering the In-Plane Structure of Metallic Phase Molybdenum Disulfide via Co and O Dopants toward Efficient Alkaline Hydrogen Evolution. *ACS Nano* **13**, 11733-11740 (2019).

20. Yin J*, et al.* Atomic Arrangement in Metal-Doped NiS2 Boosts the Hydrogen Evolution Reaction in Alkaline Media. *Angew. Chem. Int. Edit.* **58**, 18676-18682 (2019).

21. Liu S*, et al.* Dislocation-Strained IrNi Alloy Nanoparticles Driven by Thermal Shock for the Hydrogen Evolution Reaction. *Adv. Mater.* **32**, 2006034 (2020).

22. Zhu ZJ*, et al.* Ultrathin Transition Metal Dichalcogenide/3d Metal Hydroxide Hybridized Nanosheets to Enhance Hydrogen Evolution Activity. *Adv. Mater.* **30**, 7 (2018).

23. Zhou M*, et al.* Construction of Polarized Carbon-Nickel Catalytic Surfaces for Potent, Durable, and Economic Hydrogen Evolution Reactions. *ACS Nano* **12**, 4148-4155 (2018).

24. Subbaraman R*, et al.* Trends in activity for the water electrolyser reactions on 3d M(Ni,Co,Fe,Mn) hydr(oxy)oxide catalysts. *Nat. Mater.* **11**, 550-557 (2012).

25. Sheng WC, Myint M, Chen JGG, Yan YS. Correlating the hydrogen evolution reaction activity in alkaline electrolytes with the hydrogen binding energy on monometallic surfaces. *Energy & Environmental Science* **6**, 1509-1512 (2013).

26. Sheng W, Zhuang Z, Gao M, Zheng J, Chen JG, Yan Y. Correlating hydrogen oxidation and evolution activity on platinum at different pH with measured hydrogen binding energy. *Nat. Commun* **6**, 5848 (2015).
